# Synchrotron-based high-resolution photoemission spectroscopy study of ZIRLO cladding with H_2_O adsorption: Coverage and temperature dependence

**DOI:** 10.1038/s41598-020-63585-5

**Published:** 2020-04-20

**Authors:** Sangjune Park, Ki-jeong Kim, Jeongmook Lee, Jong-Yun Kim, Dong Woo Lee, Sang Ho Lim, Young-Sang Youn

**Affiliations:** 1 Department of Chemistry, Yeungnam University, Daehak-ro 280, Gyeongsan, Gyeongbuk 38541 Republic of Korea; 2 Pohang Accelerator Laboratory, Pohang University of Science and Technology, 80 Jogokro-127-beongil, Nam-gu, Pohang, Gyeongbuk 37673 Republic of Korea; 3 Nuclear Chemistry Research Team, Korea Atomic Energy Research Institute, 111, Daedeok-daero 989beon-gil, Yuseong-gu, Daejeon, 34057 Republic of Korea; 4 Radiochemistry & Nuclear Nonproliferation, University of Science & Technology, Gajeong-ro 217, Yuseong-gu, Daejeon, 34113 Republic of Korea

**Keywords:** Chemistry, Materials science

## Abstract

The coverage and temperature dependence of ZIRLO cladding with H_2_O adsorption are studied using synchrotron-based high-resolution photoemission spectroscopy (HRPES). Based on the analytical results of the Zr 3*d*, O 1 *s*, C 1 *s*, and Sn 3*d* HRPES profiles prior to H_2_O adsorption, we determine the surface compositions of O^2−^, hydroxyl OH^−^, chemisorbed H_2_O, zirconium carbide, adventitious carbon, Sn metal, and SnO_2_ in ZIRLO. When ZIRLO is exposed to H_2_O molecules, the relative proportion of zirconium metal decreases, whereas that of the total zirconium oxides increases, suggesting the reaction between H_2_O and the zirconium metal in ZIRLO. On annealing a sample with 1000 L H_2_O on ZIRLO at 300 °C, Zr_2_O_3_ and ZrO_2_ decompose, and oxygen diffuses into the bulk, thereby reducing the oxidation states of zirconium on the surface. Moreover, at this temperature, the excess H_2_O molecules on ZIRLO are thoroughly desorbed and tin element is diffused into the bulk in ZIRLO.

## Introduction

Zirconium and its alloys have been extensively used as cladding and structural materials in the nuclear industry because they possess several advantages over other materials, a low thermal neutron absorption cross-section, and good corrosion resistance, in particular^1–3^.

Cladding is mainly composed of 95% or more zirconium, and serves to completely seal the nuclear fuels in the fuel rod to prevent the external release of fission products generated from nuclear fuels by irradiation^4–7^. Several commercial claddings such as ZIRLO, ZIRCALOY-4, ZIRCALOY-2, and M5 are utilized in nuclear reactors. In order to generate electrical energy, they are always contacted with water to transfer the heat energy produced from the nuclear fuels to water^8^. Consequently, oxidation of the cladding surface by water is inevitable. Because this oxidation degrades the cladding performance, the reaction between the cladding and water should be studied to understand the oxidation phenomenon on the cladding surface.

ZIRLO, which is a commercially available cladding material, is fabricated with small quantities of niobium, tin, and iron, which are minor alloying elements, to improve the corrosion resistance and mechanical strength^3,9^. Although the oxidation of pure zirconium, ZIRCALOY-2, ZIRCALOY-4, and M5 are actively researched, studies focusing on the ZIRLO cladding are rare^1–3,8,10–18^. As ZIRLO is the most commonly used cladding material in the pressurized water reactors (PWRs), currently, investigating the oxidation behavior of ZIRLO by water is crucial to comprehend the oxidation behavior in current PWRs.

In view of the above, we performed experiments on the coverage and temperature dependence of ZIRLO cladding with H_2_O adsorption using synchrotron-based high-resolution photoemission spectroscopy (HRPES), in this study. Because synchrotron-based HRPES renders it possible to change the photon energy, we can obtain a highly surface-sensitive signal from ZIRLO based on the universal curve^19^. We determined that even before the deposition of H_2_O, the Zr 3*d* HRPES profile obtained from ZIRLO exhibited several peaks related to the Zr^0^, Zr^+^, Zr^2+^, Zr^3+^, and Zr^4+^ oxidation states, indicating that the surface of ZIRLO comprises zirconium metal and zirconium oxides. After the exposure of ZIRLO to H_2_O, the relative quantity of zirconium metal decreased, whereas the total zirconium oxide quantity including Zr^+^, Zr^2+^, Zr^3+^, and Zr^4+^ relatively increased. Sequentially, we annealed a sample with H_2_O adsorbed on ZIRLO to check the temperature dependence. On annealing up to 100 °C, there were no significant changes, whereas after annealing beyond 300 °C, the relative proportion of zirconium metal increased, and the relative quantities of the total zirconium oxides reduced with the decrease in Zr^3+^ and Zr^4+^ because of the decomposition of Zr_2_O_3_ and ZrO_2_ accompanied by oxygen diffusion into the bulk, in agreement with literature^10,20^. To the best of our knowledge, the coverage and temperature dependence of ZIRLO cladding with H_2_O adsorption have not been systematically studied using synchrotron-based HRPES.

## Materials and methods

To prepare a sample for the synchrotron-based HRPES experiments, ZIRLO (Westinghouse Electric Co.) with an area of approximately 10 × 10 mm^2^ was polished with 3000 grit SiC paper, and rinsed with deionized water. This ZIRLO sample contained 1.27 wt% niobium, 1.13 wt% tin, and 0.11 wt% iron with very small quantities of oxygen, carbon, nitrogen, and hydrogen as reported elsewhere^3^. Deionized water (H_2_O) was prepared using a Milli-Q water purification system (Millipore), and was further purified through several freeze-pump-thaw cycles to remove all the dissolved gases prior to deposition. The amount of H_2_O exposure is expressed in Langmuir (L) which is calculated as the product of the H_2_O molecule pressure and the exposure time (1 L = 10^−6^ Torr∙s).

HRPES data were obtained using the 8A2 beamline at the Pohang accelerator laboratory (PAL). In an ultra-high vacuum (UHV) chamber in which the HRPES system was installed, ZIRLO was further cleaned through five cycles of sputtering with 0.5 keV Ar^+^ ions for 1 h at 380 °C, followed by annealing at 600 °C for 30 min. Although we attempted to completely eliminate the C 1 *s* peak in the ZIRLO cladding, it continued to remain after five cycles of sputtering and annealing, similar to that reported in a previous study on ZIRCALOY-4 cladding^10,21^. Therefore, this cycle of sputtering and annealing was stopped when the intensity of the C 1 *s* peak no longer varied. All the core-level spectra of the sample with H_2_O adsorbed on ZIRLO were recorded at room temperature using a high-performance electron analyzer (SCIENTA2002, Sienta-Omicron) at photon energies of 400, 630, and 710 eV for Zr 3*d*, and 710 eV for O 1 *s*, C 1 *s*, and Sn 3*d*, where the total spectral resolution at each photon energy was 0.15 eV, determined by measuring Au Fermi-edge. HRPES data were obtained with a pass energy of 20 eV for Zr 3*d* and 50 eV for O 1 *s*, C 1 *s*, and Sn 3*d* at an energy step of 0.05 eV. The binding energies of the core-level spectra were relatively calibrated with respect to that of the 4*f*_7/2_ HRPES spectrum (84.0 eV) of clean Au for the same photon energy. The base pressure of the UHV chamber was maintained below 5.0 × 10^−10^ Torr. All the spectra were measured in the normal-emission mode, and analyzed using a standard nonlinear least squares fitting procedure with Voigt functions^22^.

## Results and discussion

**Figure 1:**
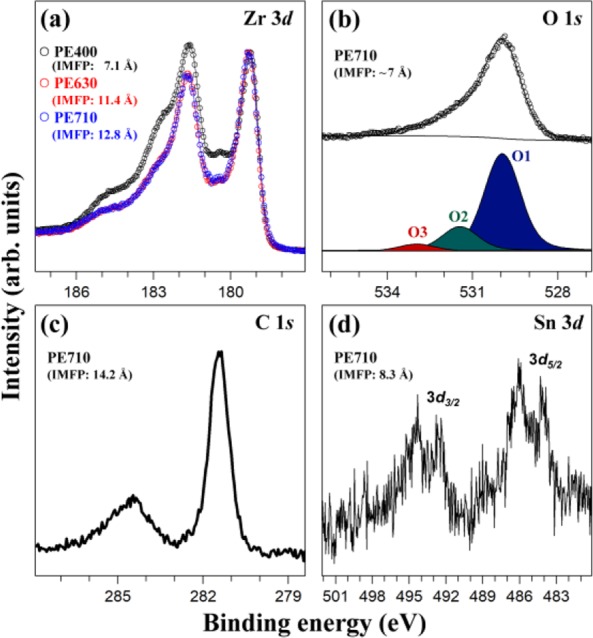
(**a**) Zr 3*d*, (**b**) O 1 *s*, (**c**) C 1 *s*, and (**d**) Sn 3*d* HRPES spectra obtained from ZIRLO before the deposition of H_2_O, where PE means the photon energy (eV). Black, red, blue open circles in (**a**) indicate Zr 3*d* spectra acquired at the photon energies of 400, 630, and 710 eV, respectively, which were normalized using Zr 3*d*_5/2_ peak of zirconium metal at 179.3 eV. The dots and the solid lines in (**b**) represent the experimental values and the peak fitting results, respectively.

**Figure 2:**
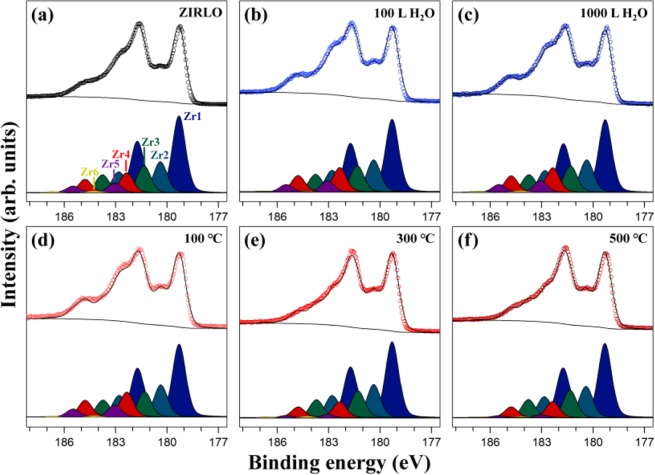
Zr 3*d* HRPES spectra of ZIRLO recorded at the photon energy of 400 eV (**a**) before the adsorption of H_2_O, after the dosing of (**b**) 100 L and (**c**) 1000 L H_2_O, and after annealing at (**d**) 100 °C, (**e**) 300 °C, and (**f**) 500 °C subsequent to the deposition of 1000 L H_2_O. The open circles correspond to the experimental values, and the solid lines are obtained by peak fitting. The experimental data in (**a**) is the same with the spectrum obtained at the photon energy of 400 eV in Fig. 1a.

Fig. 1 shows the Zr 3*d*, O 1 *s*, C 1 *s*, and Sn 3*d* HRPES spectra obtained from ZIRLO before the deposition of H_2_O. Previous study reveals that tin element alone was detected by X-ray photoelectron spectroscopy (XPS), among the minor alloying elements present in ZIRCALOY-4 cladding^10^. When we performed preliminary experiments using commercial XPS (VG Scientific ESCALAB 220i-XL), the Fe 2*p* and Nb 3*d* peaks were not observed in ZIRLO; moreover, the Nb 3*d* signal was not detected in our HRPES spectrum. Therefore, to enhance the surface sensitivity without considering the detection of the Fe 2*p* signal, we lowered the photon energy as much as possible, resulting in a photon energy of 710 eV. Furthermore, as the primary analysis in this study involves the Zr 3*d* spectra, we reduced the photon energy up to 400 eV to obtain the most sensitive Zr 3*d* HRPES profiles. Fig. 1a displays the Zr 3*d* spectra acquired at photon energies of 400, 630, and 710 eV, respectively. The Zr 3*d* profiles obtained at photon energies of 630 and 710 eV were similar; however, that obtained at 400 eV was different. The electron inelastic-mean-free-paths (IMFPs) at photon energies of 400, 630, and 710 eV for Zr 3*d*, and 710 eV for C 1 *s* and Sn 3*d* were calculated to be 7.1, 11.4, and 12.8 Å, and 14.2 and 8.3 Å, respectively, using the NIST electron inelastic-mean-free-path database (Version 1.2) with an algorithm developed by Tanuma, Powell, and Penn^23,24^. In addition, the IMFP at photon energy of 710 eV for O 1 *s* was approximately estimated as 7 Å using the universal curve^19^. In particular, the IMFPs for Zr 3*d* indicates that the spectrum at a photon energy of 400 eV contains more surface information compared to the others. Therefore, only the Zr 3*d* spectrum measured at a photon energy of 400 eV will be considered henceforth. The detailed analysis of the Zr 3*d* spectra with the peak fitting results is presented in Fig. 2.

Fig. 1b displays the O 1 *s* HRPES profiles, in which three peaks can be observed: O1 at 530.0 eV, O2 at 531.4 eV, and O3 at 533.0 eV. The O1, O2, and O3 peaks are assigned to the O^2-^, hydroxyl OH^−^, and the chemisorbed H_2_O features because the binding energies associated with them appear at 530.0–530.2 eV, 531.4 eV, and 533.4 eV, respectively, in literature^25–27^. The C 1 *s* HRPES profile is depicted in Fig. 1c, where two distinct peaks can be observed at 281.5 eV and 284.6 eV. We assigned the peak at 281.5 eV to zirconium carbide produced by the reaction between zirconium metal and the adsorbed hydrocarbon; this value is similar to the reported binding energy of 281.6–282.0 eV in previous investigations^10,28^. In addition, the peak at 284.6 eV was assigned to adventitious carbon composed of various hydrocarbon species based on the previously reported value (284.6 eV)^29^. As shown in Fig. 1c, the full width at half maximum (FWHM) of adventitious carbon at 284.6 eV is broader compared to that of zirconium carbide at 281.5 eV, which may be due to the existence of various types of hydrocarbons. In the Sn 3*d* HRPES profile (Fig. 1d), two types of Sn 3*d*_5/2_ peaks at 484.1 and 486.0 eV, and 3*d*_3/2_ signals at 492.5 and 494.4 eV, respectively, appeared with binding energy separation of 8.4 eV between them. In general, the binding energy of Sn 3*d*_5/2_ is used to analyze the Sn 3*d* HRPES profile. It is known that the binding energies of Sn metal and the SnO_2_ features in ZIRCALOY-4 cladding occur between 483.9–484.7 eV and 486.0–487.2 eV, respectively^10,18^. Therefore, we assigned the two Sn 3*d*_5/2_ peaks at 484.1 and 486.0 eV to Sn metal and the SnO_2_ compositions, respectively. Thus, by analyzing the HRPES spectra (Fig. 1) obtained from ZIRLO, we established the presence of O^2-^, hydroxyl OH^-^, chemisorbed H_2_O, zirconium carbide, adventitious carbon, Sn metal, and the SnO_2_ species.

The fact that such features appeared despite our cleaning process suggests that they may be due to the intrinsic oxygen and carbon in ZIRLO, which was also observed in prior XPS studies on ZIRCALOY-4 cladding^10,21^.

Fig. 2a depicts the Zr 3*d* HRPES profile, shown in Fig. 1a, with the peak fitting results. Because the binding energy of Zr 3*d*_5/2_ is typically utilized to analyze the Zr 3*d* HRPES profile, we explain the zirconium species using the binding energy of Zr 3*d*_5/2_. In Fig. 2a, six peaks can be observed: Zr1 at 179.3 eV, Zr2 at 180.4 eV, Zr3 at 181.3 eV, Zr4 at 182.4 eV, Zr5 at 183.1 eV, and Zr6 at 184.2 eV. We first assigned Zr1 at 179.3 eV and Zr5 at 183.1 eV to the Zr^0^ (zirconium metal) and Zr^4+^ (ZrO_2_) features, respectively, based on their binding energies ranging from 179.1–179.3 eV and 182.9–183.4 eV in literature^9,10,18,30^. In addition, it is known that the intervals of the binding energies between the Zr^+^, Zr^2+^, Zr^3+^, Zr^4+^ oxidation states are approximately 1 eV^1,31,32^. Considering the binding energy of the Zr^4+^ feature (183.1 eV) in our system, we assigned Zr2 at 180.4 eV, Zr3 at 181.3 eV, and Zr4 at 182.4 eV to the Zr^+^ (Zr_2_O), Zr^2+^ (ZrO), and Zr^3+^ (Zr_2_O_3_) compositions, respectively, which are zirconium suboxides. Moreover, Zr6 at 184.2 eV, of which a small quantity was present, was assigned to zirconium hydroxide (Zr(OH)_4_) because its reported binding energy (183.6 eV) was similar to our value^33,34^. Previously, through the analysis of the C 1 s HRPES profile in Fig. 1c, we had established the existence of zirconium carbide in ZIRLO. According to literature, the binding energy of zirconium carbide in the Zr 3*d* spectrum is 179.2 eV^10,28^, which is almost the same as that of zirconium metal (179.3 eV). Although the zirconium carbide component should be considered when the Zr 3*d* HRPES profile is analyzed, we could not confirm whether this peak was due to zirconium metal or zirconium carbide, in agreement with the previous report ^10^. Hence, we had to unavoidably ascribe the peak at 179.3 eV to zirconium metal.

It has been previously revealed that the order of the surface components in intact ZIRCALOY-4 cladding are as follows: The uppermost hydrocarbon, zirconium hydroxide, zirconium dioxide, zirconium suboxides, and zirconium bulk layers^10^. In addition, zirconium carbide is expected to exist in the hydrocarbon layer. As a result, based on the analysis of the Zr 3*d*, O 1 *s*, C 1 *s*, and Sn 3*d* HRPES spectra, along with the prior report, we concluded that the surface species in ZIRLO cladding could be the same as previously reported. Additionally, we propose that Sn metal and SnO_2_ compositions could exist within the zirconium bulk layer, and that the chemisorbed H_2_O feature could be present in the uppermost layer.

To confirm the change in the relative proportions of the zirconium features depending on the H_2_O coverage and annealing temperature, we calculated the ratio of each peak area obtained from the peak fitting results because the ratio of each peak integral in the Zr 3*d* HRPES spectra corresponds to their relative proportion (Table 1). As the proportion of zirconium hydroxide is negligible, we consider the other populations, herein. As shown in Fig. 2 and Table 1, the relative proportion of zirconium metal is the largest, and gradually decreases in the following order Zr^+^, Zr^2+^, Zr^3+^, and Zr^4+^, in accordance with their relative trends reported in literature^1^. After the exposure of ZIRLO to 100 L and 1000 L H_2_O, the relative proportion of zirconium metal decreased whereas those of the zirconium oxides including the Zr^+^, Zr^2+^, Zr^3+^, and Zr^4+^ features mostly increased (Fig. 2 and Table 1). This indicates that when H_2_O is adsorbed on ZIRLO, H_2_O and zirconium metal in ZIRLO react each other, relatively decreasing and increasing the metal population and the total quantity of zirconium oxides, respectively, in agreement with previous research on H_2_O adsorbed on pure zirconium and ZIRCALOY-2 samples^1^. This phenomenon can be explained by the dissociation of H_2_O into the adsorbed oxygen and molecular hydrogen gas on ZIRLO at room temperature as reported in the prior study of water molecule on Zr(0001)^35^. We performed annealing experiments on the 1000 L H_2_O system adsorbed on ZIRLO. As shown in Fig. 2d, the Zr 3*d* HRPES profile after annealing at 100 °C for 30 min is similar to that before annealing, indicating that any detectable change did not occur due to annealing at this temperature. However, when temperatures of 300 °C and 500 °C were applied for 30 min, the relative percentage of the Zr^0^, Zr^+^, and Zr^2+^ valence states increased, whereas those of the Zr^3+^ and Zr^4+^ oxidation states decreased (Fig. 2e,f). According to literature, on annealing at 200 °C or more, the oxidation states of zirconium are converted to lower oxidation states because of the decomposition of the Zr_2_O_3_ and ZrO_2_ compositions, and the depopulation of oxygen in the surface region accompanied by oxygen diffusion into the bulk^10,20^. Therefore, we concluded that the decomposition of Zr_2_O_3_ and ZrO_2_ and the diffusion of oxygen into the bulk lead to the reduction of the oxidation states of zirconium at 300 °C.

**Table 1:** Relative proportions of the zirconium species in ZIRLO calculated from each peak integral of the peak fitting results of Zr 3*d* HRPES spectra in Fig. 2.

	Zr^0^ (%)	Zr^+^ (%)	Zr^2+^ (%)	Zr^3+^ (%)	Zr^4+^ (%)	Zr(OH)_4_ (%)
Before deposition of H_2_O	46.4	18.5	16.3	12.0	5.8	1.0
100 L H_2_O deposition	43.7	19.0	15.7	14.5	6.7	0.4
1000 L H_2_O deposition	43.1	19.2	15.5	14.4	7.0	0.8
Annealing at 100 °C	43.1	19.0	15.0	14.8	7.2	0.9
Annealing at 300 °C	48.3	21.3	17.0	10.1	2.4	0.9
Annealing at 500 °C	48.5	20.5	18.1	10.5	1.9	0.5

**Figure 3:**
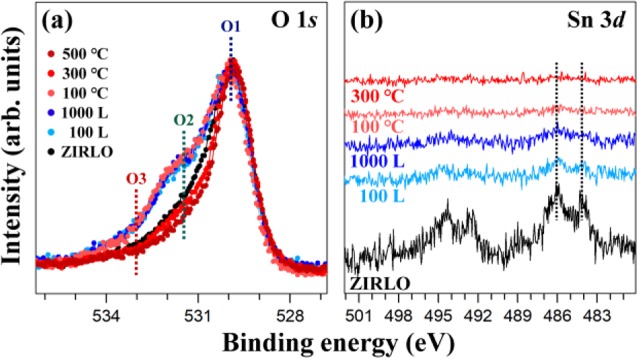
(**a**) O 1 *s* and (**b**) Sn 3*d* HRPES spectra of ZIRLO measured before the deposition of H_2_O, after the adsorption of 100 L and 1000 L H_2_O, and after annealing at 100 °C, 300 °C, and 500 °C subsequent to the deposition of 1000 L H_2_O. O 1 *s* HRPES spectra in (**a**) were normalized using O1 peak at 530.0 eV. Sn 3*d* HRPES spectra obtained after annealing at up to 300 °C are exhibited in. (**b**) The dashed lines express the positions of the binding energy of each feature.

Fig. 3 displays the coverage and temperature dependence of the O 1 *s* and Sn 3*d* HRPES profiles. As shown in Fig. 3a, after the adsorption of 100 L and 1000 L H_2_O on ZIRLO, the peak related to chemisorbed H_2_O notably increases. This increase is attributed to the excess H_2_O molecules on ZIRLO, which could be the remaining quantity after sufficient reaction with zirconium metal. After annealing at 100 °C for 30 min, the profile of the O 1 *s* HRPES spectrum remined unchanged, in accordance with the analytical result of the Zr 3*d* HRPES spectrum at this temperature. When the sample was annealed at 300 °C and 500 °C for 30 min, the enhanced peaks returned to the state before the adsorption of H_2_O on ZIRLO, indicating that the excess H_2_O molecules were completely desorbed at 300 °C. In the Sn 3*d* HRPES profiles (Fig. 3b), the peaks related to the Sn metal and SnO_2_ compositions gradually disappear due to the deposition of 100 L and 1000 L H_2_O on ZIRLO. As the adsorption of H_2_O on ZIRLO increases the surface thickness, these signals may be reduced because the probing depth for ZIRLO itself relatively becomes shallow. After annealing at 100 °C for 30 min, only the peak at 486.0 eV related to the SnO_2_ configuration remained indistinctly in the Sn 3*d* HRPES spectrum. Furthermore, after annealing at 300 °C for 30 min, it completely vanished instead of recovery, despite the desorption of H_2_O molecules at this temperature. Therefore, we infer that SnO_2_ composition decomposed and that Sn metal migrated into zirconium bulk at 300 °C, sequentially.

## Conclusions

In this study, we investigated the coverage and annealing-temperature dependence of the ZIRLO cladding with H_2_O adsorption using synchrotron-based HRPES. Through the analysis of the Zr 3*d*, O 1 *s*, C 1 *s*, and Sn 3*d* HRPES profiles obtained from ZIRLO before H_2_O exposure, we confirmed the existence of O^2−^, hydroxyl OH^−^, chemisorbed H_2_O, zirconium carbide, adventitious carbon, Sn metal, and SnO_2_ species in ZIRLO. After the deposition of H_2_O on ZIRLO, the relative proportion of zirconium metal decreased, whereas that of the total zirconium oxides increased, indicating that H_2_O reacted with zirconium metal in ZIRLO. On annealing a sample with 1000 L H_2_O adsorbed on ZIRLO at 300 °C, the decomposition of Zr_2_O_3_ and ZrO_2_ as well as the diffusion of oxygen into the bulk occurred. Furthermore, we determined that the excess H_2_O molecules were completely desorbed and that on SnO_2_ decomposition, the tin element diffused into the zirconium bulk in ZIRLO at that temperature.
